# Proteomics: a powerful tool to study plant responses to biotic stress

**DOI:** 10.1186/s13007-019-0515-8

**Published:** 2019-11-18

**Authors:** Yahui Liu, Song Lu, Kefu Liu, Sheng Wang, Luqi Huang, Lanping Guo

**Affiliations:** 1 National Resource Center for Chinese Materia Medica, China Academy of Chinese Medical Sciences, Beijing, China; 2 National Institute of Metrology, Beijing, China; 3 School of Life Science, Beijing Institute of Technology, Beijing, China

**Keywords:** Proteomics, Subcellular, Post-translational modifications, Biotic stress

## Abstract

In recent years, mass spectrometry-based proteomics has provided scientists with the tremendous capability to study plants more precisely than previously possible. Currently, proteomics has been transformed from an isolated field into a comprehensive tool for biological research that can be used to explain biological functions. Several studies have successfully used the power of proteomics as a discovery tool to uncover plant resistance mechanisms. There is growing evidence that indicates that the spatial proteome and post-translational modifications (PTMs) of proteins directly participate in the plant immune response. Therefore, understanding the subcellular localization and PTMs of proteins is crucial for a comprehensive understanding of plant responses to biotic stress. In this review, we discuss current approaches to plant proteomics that use mass spectrometry, with particular emphasis on the application of spatial proteomics and PTMs. The purpose of this paper is to investigate the current status of the field, discuss recent research challenges, and encourage the application of proteomics techniques to further research.

## Background

In the natural and agricultural environments, plants are constantly affected by biotic stress [1], which threatens their survival and growth. In response to these changing circumstances, plants have evolved a series of molecular programs to quickly perceive and adapt to the environment. The role of proteins in the plant biotic stress response is crucial because: (1) proteins participate directly in the formation of new plant phenotypes by regulating physiological characteristics to adapt to changes in the environment; (2) proteins are the critical executors of cellular mechanisms and key players in the maintenance of cellular homeostasis. However, the behavior of individual proteins usually does not reflect the complex network of signals and the dynamic regulation of cellular processes involved in the plant response to biotic stress. In fact, it is believed that the plant immune system responds to biotic stress as a complex system with interactions and crosstalk between multiple signals and with a diverse set of stress tolerance-related proteins. Therefore, many proteins are likely to function together and play multiple roles in the stress response. Currently, much of our knowledge of plant responses to biotic stress is acquired through genetic and genomic or transcriptomic approaches. Although analyses of gene and mRNA abundance have contributed greatly to our understanding of the plant immune response, the correlation between mRNA expression levels and protein levels is frequently poor [2–4]. For example, in response to *Phytophthora infestans*, nearly half of the differential proteins showed changes that not corresponded to changes in transcriptomic levels in potato [5]. Therefore, it is necessary to understand these proteins and their functions under stress at protein level.

Protein function depends not only on the molecular structure of the protein but also on its subcellular localization and post-translational modifications (PTMs) [6]. Protein function is closely related to subcellular localization because different cell components provide different physiological and biochemical environments (such as pH and redox conditions) or potential acting substrates. Most cellular biological processes and pathways involve changes in protein subcellular localization, such as the nucleocytosolic shuttling of transcription factors and relocation of mitochondrial proteins during apoptosis [7]. PTMs are a multifunctional regulatory process that can rapidly change the functional diversity of the proteome [8] and objectively reflect biological processes. Current evidence suggests that PTMs are critical for the rapid reprogramming of cells, defense signal transduction and attenuated response and are important means by which plants maintain cell homeostasis at all levels of the immune response [9].

In recent years, liquid chromatography–mass spectrometry (LC–MS) technology has become the preferred method for spatial proteomics (the localizations of proteins and their dynamics at the subcellular level) [10, 11] and PTMs because of its unique ability to measure complex protein mixtures [12–15]. LC–MS-based proteomics can show the quantitative state of a proteome [16] and contribute to unraveling cellular signaling networks and protein–protein interactions as well as provide a molecular understanding of the mechanisms involved in the response to biotic stress. Although spatial proteomics and PTMs have great development value and are technically easy to implement, (1) the diversity of the associated methods may be daunting to newcomers. (2) Our current understanding of the response of the plant immune network to biotic stress is far from comprehensive. Using these technologies to study the response of plants to biotic stress remains a research field with great potential for growth. Therefore, the purpose of this review is to summarize the latest research methods of spatial proteomics and PTMs and to describe how these methods can be used as intelligent tools to elucidate the mechanisms involved in the regulation of plant responses to biotic stress. Second, a discussion should be conducted to improve the understanding of the possibilities offered by spatial proteomics and PTMs to promote further research on plant proteomics.

## Understanding the molecular mechanisms of the plant response to biotic stress through spatial proteomics

### Subcellular separation and purification

There are great differences in protein expression in biological samples. For example, in human serum, 99% of the serum proteins consist of 22 high-abundance proteins, while the remaining 1% consist of a large number of other proteins [17]. Therefore, to easily detect a small number of target proteins or PTMs, a sufficient number of target proteins must be collected, and most of the redundant background proteins must be removed. This process requires the separation of biological samples into subcellular components or organelles to reduce their complexity. The proteins in each component are then enriched by selective fractionation, immunoprecipitation, chromatography, electrophoresis or centrifugal technologies. As a result of ongoing advances, spatial and PTMs proteomics have become tools capable of delivering answers to key biological questions, and the roles of these tools in basic and applied science will likely expand in the coming decades. Systematic efforts to map the entire or localized plant proteome depend heavily on current and emerging technologies and methods.

In recent years, spatial proteomics has become an important research field [10, 18]. The common techniques of subcellular separation and purification include centrifugation-based and affinity purification-based methods. However, the former methods are rather time consuming, and it is hard to obtain pure fractions with these methods. Meanwhile, the latter methods rely on the specificity of antibodies, and it is difficult to obtain suitable antibodies. To remedy the shortcomings of the above methods, a subcellular separation method based on different separation principles has been developed (Table 1). For instance, free flow electrophoresis (FFE) combined with two-phase partitioning to produce a population of highly purified plasma membrane vesicles has been developed. This combined high-quality plasma membrane isolation technique promoted a reproducible proteomic library of over 700 plasma membrane proteins, which were not previously identified by other studies and included peripheral membrane proteins [19].

**Table 1:** Techniques used for subcellular isolation in plant proteomic studies

Subcellular organelles/proteome	Techniques	Plant/organ	References
Cell wall proteins	Extracted procedure using sequentially CaCl_2_, EGTA and LiCl-complemented buffers	*Medicago sativa*/Stems	[20]
Apoplastic fluid	Vacuum infiltration-centrifugation	*Gossypium barbadense*/Root	[21]
Plasma membrane	Two-phase partitioning combined with free-flow electrophoresis	Arabidopsis/Seedlings	[19]
Mitochondria	Centrifugation and layered in a Percoll density gradient	*Solanum tuberosum*/Tuber	[22]
	Centrifugation combined with one-dimensional blue native polyacrylamide gelelectrophoresis	Arabidopsis/leaf	[23]
Chloroplasts	Homogenisation in sorbitol-based isolation medium with blender and centrifugation	*Malus domestica*/in vitro material	[24]
Nucleus	Flow cytometric sorting with a mild formaldehyde-based fixation	*Hordeum vulgare*/Root	[25]
Ribosome	Asymmetric flow field-flow fractionation	*Nicotiana benthamiana/*transgenic lines	[26]

The flow field-flow fractionation (FIFFF) method is an elution-based separation technique that is capable of separating biological macromolecules without relying on the sample components. Asymmetric FlFFF was employed to characterize ribosome profiles of *Nicotiana benthamiana*. With the optimized working conditions, free molecules from ribosomal subunits and intact ribosomes were separated [26]. Another promising sampling technology is laser capture microdissection (LCM), which can isolate target cell types from sectioned specimens of heterogeneous tissues via direct microscopic visualization with the assistance of a laser beam [27]. LCM has been successfully employed in rice [28].

Newly emerging proteomic methods have revealed much information at the subcellular level. These studies have led to an improved understanding of the specialized proteomic behavior of certain organs, which will help us better understand the processes of biotic stress tolerance acquisition in plants. For example, chemical proteomics provides efficiency for analyzing the proteome in a native environment. Activity-based protein profiling (ABPP) [29], engineered ascorbate peroxidase (APEX) [30], organelle-locatable reactive molecules (ORMs) [31], and proximity-dependent biotin identification (BioID) [32] have been developed for intracellular and even subcellular proteomic analysis.

### Cell wall

The plant cell wall is a supporting structure and an external physical barrier that is crucial to perceiving and limiting the stress on plant physiology [33]. Most plant cell walls are basically composed of cellulose, hemicelluloses and pectin, and some often contain polymers such as lignin [34]. During stress, plants deploy a sophisticated immune system through pattern recognition receptors (PRRs), which are biosynthesized in the endoplasmic reticulum and transported to the plasma membrane to perceive pathogen-, microbe-, and danger-associated molecular patterns (PAMPs, MAMPs, and DAMPs, respectively) and initiate pattern-triggered immunity (PTI) [35–37]. Upon pathogen attack, plants sense the pathogens and induce a defense response based on the biochemical modification of the cell wall components [38]. The pectin methyl esterification status of Arabidopsis strongly affects the resistance of this plant to *Botrytis cinerea* [39]. Furthermore, PTI can be triggered by damage associated molecular patterns, some of which are host-derived molecules from the cell wall [40, 41]. One of these types of molecules is oligogalacturonides (OGs), which are derived from homogalacturonan by pectinases and perceived by wall-associated kinase 1 (WAK1), which functions as a PRR in Arabidopsis. For resistance to *Septoria tritici* blotch, a wheat *stb6* gene encodes a conserved WAK-like protein (Stb6) that perceives the pathogen effector (AvrStb6) and confers pathogen resistance [42]. Polygalacturonase-inhibiting proteins degrade microbial polygalacturonases, resulting in a delay in plant cell pectin hydrolysis and consequently restricting fungal infection, which can be a promising strategy to sustain susceptibility to pathogens [43].

As major contributors to most of the modifications in the cell wall composition and phenotype as well as their role as receptors, perceiving fluctuations in stress and remodeling the cell wall, cell wall proteins (CWPs) play critical and dynamic roles in plants during stress [44]. To date, the largest number of CWPs identified was 805 from Arabidopsis [44], followed by *Linum usitatissimum* with 465 CWPs [45]. These data sets were obtained by combining the proteins physically located in the cell wall with proteins that are not physically located in the cell wall but are involved in biological processes related to the cell wall. Dirigent proteins (DIR) and DIR-like proteins, which were identified to be differentially expressed under stress conditions in the plant cell wall, were reported to take part in the lignin biosynthetic pathway [46, 47]. DIR-like protein-encoding genes are induced under biotic stress, such as during infection by *Physcomitrella patens* and *Pectobacterium carotovorum*. Although the large number of different DIR genes expressed during wounding and pathogen infection indicate the participation of DIR proteins in pathogen defense, direct evidence of the activity of DIR proteins and DIR-like proteins against pathogens requires techniques such as proteome studies [48]. In addition, DIR proteins could mediate the spatial control of lignin deposition, which may indicate the roles of these proteins in the maintenance of cell wall integrity.

Plasmodesmata are specialized tubular structures in the plant cell wall that allow intercellular communication and transport [49, 50]. Proteomic studies of plasmodesmata components have revealed the function and structure of plasmodesmata, and for example, 61 plasmodesmata-associated proteins were identified in Arabidopsis leaves, among which, some were responsive to stress [51]. It has been shown that intercellular communication by plasmodesmata is a MAMP-triggered immune response that results in plasmodesmata closure [52, 53]. However, some pathogens have evolved mechanisms that take advantage of plasmodesmata to spread throughout a plant. For example, *Magnaporthe oryzae* secretes cytosolic effector proteins that have been observed to move from the site of infection to other uninfected cells through plasmodesmata [54]. Another case was found in *N.benthamiana* in response to turnip mosaic virus, in which a plasmodesmata-specific cell wall-loosening protein called NbEXPA1 was downregulated by viral infection. It is believed that this plasmodesmata protein was recruited by viral replication to promote potyviral infection [55].

Though bioinformatic methods provide a launch pad to characterize CWPs with high-throughput proteomic profiles, there remain proteins annotated as having unknown functions and classified as ‘hypothetical’ by bioinformatic methods. The combination of morphological approaches and plant cell wall proteomics, which are supported by the analysis of the three-dimensional organization of CWPs, has great potential to provide information for understanding the function of the cell wall in plant immunity.

### Apoplast

Apoplast is the space outside the plasma membrane and comprises the cell wall matrix and the fluid in the intercellular spaces, that allows free movement of material. The fluid moving in the extracellular space is usually named apoplastic fluid. It contains a large variety of molecules and proteins that are known to be involved in many biological processes [56]. The most commonly used method to collect apoplastic fluid is vacuum infiltration-centrifugation (VIC), the method can be used for the analysis of the roots, stems and leaves [57, 58]. Briefly, the tissues are infiltrated with a buffer during vacuum conditions, and the infiltrated apoplastic fluid is collected by centrifugation. In VIC application, centrifugal and buffer are two key factors. It is reported that the centrifugal forces below 1000 g lead to get pure apoplastic fluid [59]. In addition, the number of proteins collected by using 100 mM Sodium phosphate buffer [60], 0.2 M CaCl_2_ buffer [58] and buffer with different ionic strength [61] was significantly different. Apoplastic fluid can also be collected by direct centrifuging, for example, in *Beta vulgaris* it was extracted by direct leaf centrifugation [62]. At present, about 600 proteins have been identified in apoplastic fluid [63]. The functions of these proteins are mainly involved in general metabolism, polysaccharide metabolism, proteolysis, oxido-reductase and plant defense [57].

Plant pathogenic bacteria have different adaptability to their parasitic environment. For example, phytopathogenic pseudomonads exhibit a different response according to their living on the surface and the interior of leaves [64]. This indicates that, for these bacteria, adaptation to apoplast environment is the key to establish parasitic lifestyle and spread [65]. However, when plants under biotic stresses, a large number of pathogenesis related proteins and disease resistance proteins containing leucine-rich repeats (LRR) domain, which frequently increase in apoplastic fluids [57].

Moreover, exosomes, which are extracellular vesicles (EVs), play a critical role in intercellular signaling in mammals by transporting proteins and small RNAs, as well as in responding to pathogen infection in plants. Proteomic analyses of EVs from the apoplastic fluids of Arabidopsis revealed that these vesicles are highly enriched in proteins involved in biotic stress responses. Consistent with this finding, EV secretions were enhanced in plants infected with *Pseudomonas syringae*, in which many proteins, such as RPM1-interacting protein4 (RIN4) and the glucosinolate transporter PEN3, were highly induced in response to stress [63]. These findings reveal that EVs may represent an important component of the plant immune response.

### Plasma membrane

The plasma membrane consists of a phospholipid bilayer with embedded proteins that separates the cell interior from the external environment. Approximately half of the membrane volume is membrane proteins. As a communication interface with the extracellular environment, significant intracellular restructuring of the plasma membrane can be caused by biotic and abiotic stress in plants [66]. Thus, proteomic studies on plasma membrane proteins are essential for exploring pathways of signal transduction and elucidating the mechanism of the plant defense system. The commonly used procedure to analyze the plasma membrane proteome is to first isolate the membrane fraction through centrifugation [67]. Most known PRRs are present at relatively low levels in the plasma membrane; however, it has also become possible to perform high-throughput, systematic measurements of the proteomes of plasma membranes. It has been reported that more than 3900 proteins were observed in highly purified rice plasma membranes [68]. Plant membrane proteins, especially plasma membrane-localized receptor-like kinases (RLKs) and receptor-like proteins (RLPs), which function as pattern recognition receptors (PRRs), play a central role in adaptation to biotic stress. Usually, a plant RLK contains an extracellular domain, a single pass transmembrane domain and an intracellular kinase domain [69], whereas an RLP is essentially an RLK lacking an intracellular kinase domain.

Many of these RLKs can determine pathogen perception as well as propagate signals downstream of pathogen recognition [70, 71]. The classic example, which is the most characterized PRR-elicitor pair in Arabidopsis, is the flagellin sensing2 (FLS2)-brassinosteroid insensitive1-associated kinase1 (BAK1) receptor complex that can perceive a 22-amino-acid epitope (flg22) of the bacterial flagellin. Cys-rich receptor-like kinases (CRKs) are a large subfamily of RLKs in Arabidopsis [72]. A subset, CRK28, was demonstrated to be associated with BAK1; together, these proteins form the immune receptor complex. In this model, CRKs showed upregulation upon the perception of flagellin and all acted to enhance the plant immune response to *Pseudomonas syringae* [73]. Pathogen elicitors, such as small molecules from insect saliva, may be recognized by the somatic embryogenesis receptor kinase (SERK)/BAK1 and trigger defensive signaling pathways further downstream in host plants [74]. According to proteomics data, infestation by the pea aphid caused SERK to stimulate PTI defenses in A17 plants, which were isogenic *Medicago truncatula* genotypes without the R gene. However, for Jester plants, which contain the R gene, aphid infestation did not trigger SERK, and the PTI defenses were lower than those in the A17 plants [75].

### Mitochondria

Mitochondria play a vital role in plant cells. These organelles are not only involved in pathways for energy production and signal transduction but also play roles in cellular metabolism and programmed cell death (PCD). According to bioinformatic predictions, there are up to 3000 proteins present in mitochondria in plant cells under certain stages or conditions [76]. However, even for energy-related proteins, which are the most thoroughly studied functional protein group in mitochondria, less than 75% of the proteins are recognized as mitochondrial by one prediction algorithm [77]. A recent proteomic analysis showed that the plant mitochondrial proteome is well defined and probably consists of 2000 or more different types of proteins [23, 77].

During metabolic processes, the mitochondrial electron transport chain (mETC) will produce reactive oxygen species (ROS) as a signal in response to biotic stress [78]. A large amount of research has indicated that ROS levels increase under various forms of stress, such as extreme temperature, drought and pathogen stress, through changes in oxidative phosphorylation systems [79–83]. Some proteins related to metabolic processes could be coinduced in response to nonhost pathogens. For example, a proteomic study showed that the ability of soybean to respond to *Bipolaris maydis* stress depends on several metabolic proteins, such as oxygen evolving enhancer (OEE) and mitochondrial processing peptidase (MPP) [84]. In addition, autophagy-mediated mitochondrial degradation (mitophagy) has important effects on the normal function of mitochondria upon pathogen exposure [82]. In one iTRAQ-based proteomic study, wild-type Arabidopsis plants and autophagy-deficient mutant plants were analyzed upon exposure to *Verticillium dahlia*. In this case, the authors proved that autophagy is involved throughout the defense process and is required for activating defense responses; autophagy-mediated mitochondrial degradation also occurs [82].

Increasing evidence has demonstrated that mitochondria participate in PCD [85–89]. The rice dynamin-related protein 1E negatively regulates plant PCD by modulating the mitochondrial structure and cytochrome c release. Heat shock protein (HSP) family induction under stress to prevent protein misfolding and irreversible protein aggregation is also involved in the PCD process [90, 91]. These findings suggest that respiratory homeostasis is important to enhance tolerance to stress and that activation of the defense response system may help strengthen plant stress resistance.

### Chloroplasts

The plant chloroplast apparatus not only plays an important role in conducting photosynthesis but also participates in many biochemical processes, such as sensing environmental stimuli and synthesizing pigments and plant hormones [92]. The isolation and purification of chloroplasts from different organs are significant steps in profiling the chloroplast proteome. Most of the proteins located in the chloroplast are covered with a membrane, so it is difficult to analyze these proteins by gel-based proteomic techniques; however, this issue can be resolved by a method involving protein extraction with organic solvents [93]. Intact chloroplasts of wheat have been isolated from leaves [94].

Increasing evidence has revealed the central role of chloroplasts in the plant stress response [95–97]; chloroplasts act as environmental sensors by mediating the plant stress response and redox sensor activation and by coordinating nuclear-encoded plastid-localized proteins [98–100]. Chloroplasts are involved in the biosynthesis of phytohormones such as salicylic acid (SA) and jasmonic acid (JA), which are the most prevalent defense phytohormones produced in response to biotic stress. In addition, research suggests that phytopathogens target chloroplast homeostasis as a pathogenicity mechanism [101]. Recently, the chloroplast retrograde pathway was demonstrated by proteomics to regulate the plant immune response by affecting the glucosinolate pathway and SA-/JA-mediated signaling pathways [99, 102]. The jasmonate ZIM-domain 7 (JAZ7) protein generally functions as a transcriptional repressor regulating various biological processes, such as increasing the susceptibility of Arabidopsis to the fungal pathogen *Fusarium oxysporum* [103], and this protein is also involved in the defense response against *Pseudomonas syringae pv. tomato* DC3000 (PstDC3000) via regulation of ROS, the energy balance and glucosinolate biosynthesis [104].

Chloroplasts are another type of ROS factory in cells, so these organelles are also sensitive to ROS signaling. Many ROS defense systems are active under stress, such as guaiacol peroxidase-mediated ROS scavenging [105], DC90-mediated ROS scavenging [106] and antioxidant systems [107, 108]. Maintenance of high photosynthetic performance also helps enhance stress tolerance [109]. The HSP family is important for maintenance of the normal function of photosynthesis under temperature-related stress [110] and is involved in abscisic acid (ABA) signal transduction [111].

### Nucleus

The nucleus contains most of the genetic information and initiates transcription for protein expression. Therefore, under biotic stress, the nucleus determines how the plant will respond to external stimuli. Proteins, as the most abundant nuclear components, cooperate with nucleotide polymers and play vital roles in the nucleus. Furthermore, there is a complex network of mechanisms that respond to stimuli inside the nucleus. Altogether, the proteome of the nucleus requires further study. Recent advances in nuclear proteomic methodologies have allowed highly sensitive identification of nuclear proteins [112, 113]. For example, approximately 4975 nuclear proteins in leaves were detected in soybean, many of the identified proteins among which belonged to nuclear localization signals and were homologs of transcription factors and other nuclear regulatory proteins [114].

Plant cell nuclei can sense signals from pathogens and translate these signals into molecular responses, which act as warnings to allow the early detection of an impending pathogen assault, notably through rapid modulation of the proteome [115]. Dramatic changes in the nuclear proteome of *Solanum lycopersicum* upon *Phytophthora capsici* infection were reported [116]. The AT-hook-like (AHL) protein family was found to contribute to immunity. The AHL1 and AHL9 proteins accelerate PTI responses, suggesting that modification of AHL protein abundance or function could be employed to enhance disease resistance. When apple was exposed to *Venturia inaequalis*, the nuclear proteome of apple leaves was mainly involved in ROS scavenging and ubiquitin proteasome-mediated protein degradation [117].

### Vacuoles

The vacuole is the largest organelle in plants that can accumulate inorganic ions and metabolites, store nutrients and degrade discarded macromolecules or organelles [118]. Vacuoles can detoxify the cell by using ‘internal excretion’, store the modified toxins, improve plant salinity tolerance via intracellular Na^+^ compartmentalization and defend against biotic stress by storing large amounts of secondary metabolites [119]. Vacuoles can remove toxic H_2_O_2_ by vacuolar peroxidases [120] and receive damaged proteins or organelles by autophagy [121]. These results show that vacuoles may be involved in toxic salt accumulation, osmotic regulation, ROS scavenging and mitophagy to defend against many biotic stress factors.

Proteins exhibit spatiotemporal changes (that is, changes in location and expression), which enables the cell to adapt to biotic perturbations and form the proteome network, thereby defining the cell’s function and phenotype. Although we are witnessing noteworthy progress in the understanding of the subcellular proteomes of various plants, it should be noted that a few thousand proteins that have been identified in the plant proteomes have not been functionally described. In practice, it has been challenging to characterize the full potential of certain proteins based on abundance, type, number or localization. Thus, for this field, we believe that the potential for an increased number of future discoveries in plant cell biology is tremendously high.

## Recognizing PTMs involved in plant defense

Proteins can be further post-translationally modified by covalent addition of some chemical units or by changing the structures of the amino acids themselves. Therefore, no whole proteome is complete without a map of PTMs. PTMs are regulatory processes that can affect protein structure, function, subcellular localization, activity and stability, which play critical roles in almost all biological processes [122, 123]. PTMs are often significant molecular engine that can lead to tremendous changes in the regulation of biological processes even when there are no changes at the total protein or transcript levels [124]. Currently, there are more than 400 known PTMs reactions, and this number is increasing [125]. PTMs proteomics is a fast-growing field that aims to provide a comprehensive analysis of protein PTMs and a better understanding of the regulatory roles of protein PTMs in molecular networks and ultimately the global impact of these PTMs on plant biological processes.

### Post-transcriptional modification enrichment

Although PTMs play an extremely important role in numerous cellular processes, the analysis of PTMs remains challenging because of their complex structures, dynamic nature and low abundance. In addition, repeatability and noninvasive enrichment methods are essential for biological studies. Fortunately, substantial progress has been made in developing and optimizing enrichment and fractionation strategies for global studies of PTMs (Table 2). The enrichment strategies can be applied to the protein level or the peptide level. Peptide-based enrichment strategies are by far the feasible methods. A brief introduction of the various enrichment strategies has been provided in this paper. For phosphorylation research, the most productive approach is based on exploiting metal chelation. Immobilized metal affinity chromatography (IMAC) and metal oxide affinity chromatography (MOAC) methods are typical examples of efficient ways to enrich phosphorylated peptides from complex mixtures based on the interaction between negatively charged phosphate groups and positively charged metal ions or metal oxides. Enrichment with TiO_2_ beads has become a routine method in plant proteomic studies in more recent years [126]. Another efficient and sensitive method for the enrichment of histidine-phosphorylated peptides using Fe^3+^-IMAC columns was established, as well as an optimized sample preparation strategy, which allows the quantification of all phosphorylation events and requires less input samples than other methods [127].

**Table 2:** New methods and technologies for the identification of PTMs of proteins

PTMs	Method and technology	References
Phosphorylation	Polymer-supported metal-ion-affinity capture	[128]
	Functional ligand-binding identification by Tat-based recognition of associating proteins	[129]
Glycosylation	Dendrimer-conjugated benzoboroxole	[130]
	Activated ion electron transfer dissociation	[131]
Ubiquitination	Combined fractional diagonal chromatography	[132]
Sumoylation	Replaced SUMO1 and SUMO2 isoforms with a variant and purification	[133]
Acetylation	Peptide prefractionation, immunoaffinity enrichment	[134]
Methylation	Combining SCX, IMAC and H-pH-RPLC	[135]

*SCX* strong cation exchange, *IMAC* immobilized metal ion affinity chromatography, *H-pH-RPLC* high-pH reversed-phase liquid chromatography, *SUMO1/2* small ubiquitin-like modifier isoform 1/2

Glycosylated proteins produce greater proteome diversity than any other PTMs. To date, there has been a lack of methods for the analysis of complete glycosylated peptides at the whole-proteome scale [136]. For glycosylation, many methods have also been employed for the enrichment of glycosylated peptides/proteins, including lectin affinity chromatography [137], hydrazide chemistry [138], and hydrophilic interaction liquid chromatography (HILIC) [139]. A total of 971 O-glcNAc-modified peptides were identified using lectin weak affinity chromatography in one proteomic study in Arabidopsis [137]. In addition, 1152 *N*-glycopeptides were identified from Arabidopsis inflorescence tissue by wheat germ lectin weak-affinity chromatography [140].

To study plant protein ubiquitination, in vitro ubiquitination assays are often employed and have been demonstrated to be one of the most suitable tools for testing the functions of ubiquitin substrates [141]. However, purified proteins, especially those with high solubility and expression levels, are not always easy to analyze by this method. The Arabidopsis ubiquitination profiling in *Escherichia coli* was reconstituted using a synthetic biological approach. In this system, the plant proteins are expressed and then immediately participate in ubiquitination reactions within *E. coli* cells. Additionally, this system allowed the efficient purification of ubiquitin conjugates in milligram quantities [142]. Recently, a sensor-based proteomic approach that takes advantage of the Vx3K0-Lysine63 polyubiquitin specific sensor was used [143]. Vx3K0-Lys63 is based on three repetitions of ubiquitin interaction motifs from yeast that are joined by lysine63-linked polyubiquitin chains [144]. Combined with LC–MS/MS, this approach identified over 100 proteins modified with Lys63 polyubiquitin in Arabidopsis [143].

There has been an increasing interest in plant PTMs research, yet studies hardly examine multiple PTMs at one time. The main barriers of PTMs studies are the requirement of large amounts of protein and the time consuming of sample preparation. Therefore, proteomic quantification of multiple PTMs by a new method is required. For example, the “one-pot” PTMs enrichment approach makes it possible to identify and quantify the peptides containing acetylated and succinylated lysine residues from 1 mg of mitochondrial protein sample [145].

### Phosphorylation

Phosphorylation of serine, threonine and tyrosine residues has been widely demonstrated, but the degree of histidine residue phosphorylation is low, and histidine phosphorylation is considered an archaic type of protein phosphorylation. Based on large-scale proteome analyses of plants, phosphorylation was shown to play essential roles in the regulation of nearly all biological phenomena, including proliferation, differentiation, apoptosis, and cellular communication [146].

In Arabidopsis, two classic PRRs, namely, FLS2 and EF-Tu receptor (EFR), which recognize bacterial flg22 and the EF-Tu epitopes (elf18), respectively, both associate with BAK1 [also known as somatic embryogenesis receptor kinase 3 (SERK3)] in a ligand-dependent manner. FLS2-BAK1 interacts with botrytis-induced kinase 1 (BIK1) to initiate plant immunity, which is based on BIK1 phosphorylation by BAK1 [1]. Similarly, PTI can also be suppressed by counteracting the kinase activity by serine/threonine protein phosphatase 2A (PP2A) activity. Upon flg22 treatment, PP2A activity has to be sacrificed for the activity of BAK1 and PTI signaling [147]. Additionally, protein phosphatase PP2C38 interacts with FLS2-BIK1, acts as a negative regulator of BIK1 and inhibits BIK1 phosphorylation. A combined approach using coimmunoprecipitation (Co-IP) and phospho-proteomic analyses demonstrated that activated BIK1 induces phosphorylation of PP2C38 at Ser-77, which dramatically decrease PP2C38 activity and its interaction with the FLS2/BAK1 or EFR/BAK1 receptor complexes [148]. The remarkable interactions between immune regulatory kinases and phosphatases are a significant mechanism by which plant cells achieve homeostasis at the proteome level during infection.

Phosphorylation dynamics are essential for the regulation of immune responses. Many bacterial pathogens inject effector proteins into the host cell via the type III secretion system (TTSS). Thus, there has always been a struggle between host cell immune responses and pathogen effectors. HopAO1, which is a tyrosine phosphatase, is delivered by PstDC3000. Once HopAO1 is ectopically expressed in Arabidopsis, it inhibits elf18- and flg22-induced ROS bursts and resistance to PstDC3000, which is part of its virulence strategy [71]. HopAO1 expression led to an ~ 50% reduction in the phosphorylation of EFR upon elf18 treatment. This reduction was partially dependent on HopAO1 catalytic activity, confirming that a major virulence function of HopAO1 is the targeted dephosphorylation of PRRs with the purpose of inhibiting MAMP-induced immune responses [71].

### Ubiquitination

The ubiquitin system typically involves the covalent attachment of a highly conserved 76-amino-acid small protein, ubiquitin, to the ε-amino group of a lysine residue (Lys) of a substrate protein. Ubiquitination is reversible, ATP dependent, and catalyzed by the ubiquitin-activating enzyme (E1), ubiquitin-conjugation enzyme (E2), and ubiquitin ligase (E3) cascade [149]. To date, one E4 ligase mutant, snc1-enhancing3 (MUSE3), has been identified in Arabidopsis [150]. Ubiquitination can produce monoubiquitinated or polyubiquitinated proteins, chains of which can be formed by linking more than one lysine residue (e.g., Lys11, Lys48, Lys63) to ubiquitin. The vital roles of ubiquitination are responsible for the selection, targeting and proteolysis of specific substrates destined for degradation [151, 152].

The ubiquitin proteasome system participates in plant immunity regulation via PRRs that are ubiquitinated and targeted for degradation in the ubiquitin proteasome system. For example, two ubiquitin E3 ligases containing plant U-box (UPB) domains, PUB12/13, are phosphorylated by BAK1, which is required for FLS2-PUB12/13 association. Then, PUB12/13 polyubiquitinate FLS2 and promote the degradation of this protein to attenuate immune signaling [153]. In addition, AtUBP12 and its solanaceous ortholog NtUBP12, which are two deubiquitinating enzymes, were both identified as negative regulators of HR, indicating that these proteins participate in regulating plant immunity against virulent PstDC3000 in Arabidopsis [154]. Two rice seedling samples were investigated by label-free quantitative proteomics after treatment with flg22 and chitin [155]. The ubiquitination levels of some key components in the phenylpropanoid metabolic pathway were upregulated in plants; however, the ubiquitination levels of many enzymes in the plant hormone signaling pathways were up- or downregulated. This finding suggests that ubiquitination may fine tune hormone pathways for defense responses.

### Sumoylation

Small ubiquitin-like modifier (SUMO) is an ~ 100-amino-acid polypeptide that is covalently attached to target proteins in a process resembling the conjugation of ubiquitin. SUMO has 3 active isoforms, SUMO-1, -2 and -3, with mature SUMO-2 and -3 having 97% identical sequences [156]. Notably, SUMO-1 and -3 themselves can be further modified by SUMO, which results in polySUMO chains. Sumoylation is directed by an enzymatic cascade analogous to ubiquitination, and SUMO is generally conjugated onto lysine residues of target proteins [157]. Currently, the number of high-confidence SUMO targets in Arabidopsis is 1058, most of which are nuclear localized [133]. Mature SUMO is conjugated to substrates by the SUMO-activating enzyme (SAE or E1), SUMO-conjugating enzyme (SCE or E2), and SUMO ligase (E3), which can promote sumoylation [158]. Two SUMO E3 ligases were identified in Arabidopsis: SIZ1 and high ploidy 2 (HPY2, also known as AtMMS21). Notably, the level of sumoylation detected in SUMO targets is often low, with less than 10–20% of the targets being modified; however, SUMO attachment appears to affect the function of the entire pool of a target protein [159]. This fact implies that SUMO attachment regulates target functions by affecting some processes, such as cell cycle progression and protein stability, rather than by activating its targets per se [160].

Sumoylation is essential for both normal cellular functions and stress defense and is especially critical for pathogen growth, conidium formation and virulence of the host cell. With the combination of a proteomic approach and biological phenotypic analyses, the deletion mutants of the sumoylation pathway genes *AOS1* and *UBA2* (belonging to E1) and *UBC9* (E2) and *SIZ1* (E3) were researched [161]. During infection by the rice blast fungus *Magnaporthe oryzae*, these genes were all significantly reduced in strength, and the mutants exhibited inhibition of host penetration and invasive growth [161]. A total of 940 predicted SUMO proteins were identified, most of which were related to conidial storage accumulation and mobilization, cell wall construction, stress response, redox detoxification, cell cycle control, and signal transduction [161]. Notably, this study demonstrated that all core components of septins are SUMO targets and that septins are sumoylated in *M. oryzae*, indicating that sumoylation of septins can regulate the function of septins by affecting the location of septins in the appressorial septin ring and thus is important for infection [161].

### Acetylation

Protein acetylation is a prevalent protein modification that mainly occurs on the N-terminal (Nt) or lysine residue. Nt-acetylation is catalyzed by Nt-α-acetyltransferases (NATs), which the acetyl moieties are transferred from acetyl-CoA to the α-amino group of the Nt-residue [162]. Even if the fact that more than 80% of proteins in humans and plants are estimated to be Nt-acetylated [163, 164], its functional and significance are still enigmatic. Notably, it has been discovered that Nt-acetylation may be dynamic, working as a regulator in plant immunity. For example, Nod-like receptors (NLRs), the stability is of which is tightly regulated, function as immune receptors in plants. Overaccumulation of NLRs often results in autoimmunity, whereas NLR deficiency can cause susceptibility to specific pathogens [165]. One NLR protein, suppressor of natriuretic peptide receptor A (NPR1), constitutive 1 (SNC1), is regulated by different NATs. NAT complex A (NATA) contributes to the first methionine (Met) acetylation of SNC1, while the second Met is acetylated by NAT complex B (NATB). Proteomic analyses revealed remarkable results, showing that Nt-acetylation of NATA and NATB have opposite effects on protein abundance and that NATA destabilizes SNC1 and is identified as a modulator of SNC1-mediated responses to pathogens. However, NATB stabilizes SNC1. SNC1 protein accumulates in NATA mutant plants and enhances pathogen tolerance [165].

In contrast to Nt-acetylation, lysine acetylation is a reversible and dynamic post-translational modification that is catalyzed by histone acetyltransferases (HATs) or histone deacetylases (HDACs) on the histone tails. The latest research by MS-based technology in maize leaf identified 2791 acetylation sites [166], which is twice the number detected in a rice acetylome study (1337 acetylation sites) [167]. It is obvious that host proteins can directly be acetylated by pathogen effector proteins encoding HATs to alter immunity. For instance, general control nondepressive 5 (GCN5) and alteration/deficiency in activation 2 (ADA2) are known as two subunits of the HAT complex, which acetylates histones (H3K9) and functions in immune signaling pathway by activating the expression of defense-related genes. The cytoplasmic effector PsAvh23 is produced by the soybean pathogen *Phytophthora sojae*. PsAvh23 disrupts the ADA2-GCN5 complex by binding to ADA2 and further suppresses H3K9 acetylation mediated by the ADA2-GCN5 module, promoting *P. sojae* infection [168]. Moreover, endogenous plant enzymes can regulate protein acetylation during the immune response. The maize pathogen *Cochliobolus carbonum* produces the HC-toxin (HCT) as an HDAC inhibitor. A new study based on iTRAQ proteomics with an immobilization strategy has been conducted in the quantitation of protein acetylation in HCT-treated or pathogen-infected plants. These studies uncovered that HCT is required for infection and plays an important role in altering activity of histone deacetylases, which further influence both histone and nonhistone protein acetylation during a plant–pathogen interaction [166].

### Glycosylation

Glycosylation of proteins is one of the most abundant modifications found in the proteomes of higher eukaryotes. The most prevalent complex type is asparagine (N)-linked glycosylation of proteins, which includes several modifications, including β-1,2-linked xylose (Xyl), core α-1,3-linked Fuc, and Lewis-A epitope structures. In addition to N-glycosylation, other types of protein glycosylation have been reported in plants, such as a single N-acetylglucosamine on Ser/Thr residues (O-GlcNAcylation) and glycosylphoshatidylinositol (GPI) anchors on the C-termini of proteins [169, 170].

Plants use immune receptors to recognize pathogen effectors and activate effector-triggered immunity (ETI); glycosylation of receptors and/or effectors can regulate immunity processes. For instance, *Meloidogyne graminicola* has evolved a novel effector, MgGPP, that is secreted into rice cells; this effector targets the endoplasmic reticulum (ER) and is upregulated in the early parasitic stage of *M. graminicola*. Notably, N-glycosylation of MgGPP is required to suppress the host response [171]. Another example is the glycosylation of flagellin. With the use of matrix-assisted laser desorption ionization-time-of-flight MS (MALDI-TOF MS) analysis, a ten-gene cluster of *gigX* genes, which are glycosylation island genes of *Xanthomonas oryzae pv. oryzae,* was proven to determine flagellin glycosylation, resulting in the regulation of motility and virulence of *X. oryzae pv. oryzae* [172]. SA homeostasis was also studied by proteomics. UGT76D1, a unique uridine 5′-diphospho-glycosyltransferase, plays a vital role in SA homeostasis and is associated with the immune response to PstDC3000 in Arabidopsis. After UGT76D1 expression, the formation of dihydroxybenzoic acid (DHBA) glycosides is accelerated. In addition, DHBA glycosylation may activate and increase SA synthesis. High levels of SA accumulation lead to oxidative bursts and R protein expression and ultimately to PCD and increased plant resistance to PstDC3000 [173].

### Other PTMs

Protein carbonylation is the direct oxidation product of proline, lysine, arginine and threonine [174, 175]. Lysine residues are most sensitive to carbonylation and are considered to be markers of protein oxidation [176]. This PTMs leads to the loss of protein function and ultimately to the degradation of oxidized proteins [177]. The early response of plants to biotic stress is usually associated with oxidative bursts that leads to carbonylation of proteins. For example, pathogens can cause a significant increase in the carbonylation of proteins in plants [178]. In addition, the identification of oxidized amino acid residues in proteins may provide important information for the oxidation mechanism and metabolic pathway of cell vitality loss under stress [179].

Some PTMs perform immunomodulation, for example, phosphorylation, ubiquitin and sumoylation [180]. Although it is very important, identification of the kinase upstream of a known substrate remains a major challenge. The application of proximity-dependent affinity labeling of protein complexes and targeted quantitative proteomics is a promising strategy for the discovery of unknown kinases [181–183]. Furthermore, our understanding of other PTMs, such as S-nitrosylation and sulfenylation, and the importance of these modifications for plant immune responses is increasing [9]. An in-depth understanding of the mechanisms via which PTMs promote an effective immune response will provide a dynamic and comprehensive perspective on plant immunity and provide new strategies for improving the performance of plant responses to biotic stress.

Plant immunity mechanisms can be viewed to be the products of thousands of proteins acting with a common plan to shape the cellular response. At the cellular level, proteins function in certain environments, such as organelles, which provide specific chemical environments and a set of interaction partners that are necessary to enable the protein function [184]. The combination of proteomics techniques and the isolation and purification of organelles has produced a considerable synergy and has provided a wide range of possibilities for understanding the spatial distribution of proteins at the subcellular level. The direct identification of proteins in subcellular compartments via LC–MS-based technology remains the most popular high-throughput approach for crude and compartment-enriched samples [185]. Some proteomic studies have led to the identification of a considerable portion of the subcellular proteome of plants [186–188]. This information has led to further research on organelle function under biotic stress conditions. This is often a key step in understanding the underlying molecular mechanisms of plant resistance. For instance, plant chloroplasts [189, 190] and nuclei [191] have emerged as targets of pathogen effector proteins. Additionally, chloroplast-nucleus communication has been found to be associated with the plant immune response [192]. Accurate analyses of the subcellular localization of proteins is therefore essential for understanding plant immunity.

## Comparative protein expression in response to biotic stress

### Quantitative technology

With the rapid development of sample pretreatment and MS-based proteomic technology, qualitative proteomic analysis is becoming increasingly precise, providing increased coverage and consistent quality, rather than merely identifying proteins. The field has shifted from protein identification to accurate and reliable quantitative analysis. Because mass spectrometry itself cannot achieve quantification, numerous strategies have been developed to achieve quantification by mass spectrometry. These strategies can be distinguished from each other by their method of quantitation, i.e., labeling-based and label-free quantitation. Labeling-based strategies include isotope-coded affinity tag (ICAT), isobaric tags for relative and absolute quantification (iTRAQ), tandem mass tag (TMT), amino acid-coded mass tagging/stable isotope labeling with amino acids in cell culture (AACT/SILAC), uniform ^15^N/^18^O labeling and peptides or quantitative concatemer (QconCAT). Label-free techniques include sequential windowed acquisition of all theoretical fragment ion mass spectra (SWATH). Although these myriad approaches can be considered a bridge between quantitative and qualitative analyses, there are many choices for practical application, and each of these strategies has its advantages and disadvantages (Table 3).

**Table 3:** Comparison of main label-based proteomic quantitative techniques

Quantification technique	Labeling level	Multiplexing capability	Reaction localization	Amino acid residue	MS^n^	Characteristic	References
^15^N labeling	Protein	2-plex	In vivo and in vitro	All	MS1	Expensive; need metabolically active cells	[193]
SILAC	Protein	5-plex	In vivo and in vitro	Lys, Arg	MS1	Applicable to active cells; arginine can be converted into proline during cell division	[193]
CTAP	Protein	2-plex	In vivo and in vitro	Lys	MS1	Expensive; low multiplexing capability; need stable expression of exogenous enzymes	[194]
^18^O labeling	Peptide	2-plex	In vitro	C-terminal	MS1	Enzyme-mediated back-exchange of ^18^O with ^16^O	[195]
ICAT	Protein/peptide	2-plex	In vitro	Cys	MS1	Does not support labels without cysteine-containing peptides	[196]
ICPL	Protein/peptide	4-plex	In vitro	Lys	MS1	Support clinical samples; need complex computational analysis	[197]
TMT	Peptide	10-plex	In vitro	N-terminal, Lys	MS2	Expensive; wide application range	[198]
iTRAQ	Peptide	8-plex	In vitro	N-terminal, Lys	MS2	High throughout, strong stability; expensive	[199]
DiLeu	Peptide	12-plex	In vitro	N-terminal and *ε*-amino group of the lysine side chains	MS2	Wide application range	[200]
IPTL	Peptide	3-plex	In vitro	Employs SA (for N-terminal) and dimethyl (for C-terminal lysine) tagging	MS2	Wide application range	[201]

*SILAC* stable isotope labeling with amino acids in cell culture, *CTAP* cell-selective labeling with amino acid precursors, *ICAT* isotope-coded affinity tag, *ICPL* isotope-coded protein labeling, *TMT* tandem mass tag, *iTRAQ* isobaric tag for relative and absolute quantification, *DiLeu N*,*N*-dimethyl leucine, *IPTL* isobaric peptide termini labeling

A mass defect-based four-plex data-independent acquisition strategy (MdFDIA) was researched. In this approach, cells are grown in culture media supplemented with the isotopes ^13^C_6_^15^N_2_-lysine and D_8_-lysine. After labeling has been achieved, these two labeled protein samples were digested with Lys-C, peptides were labeled in vitro with light (2^13^CD_2_H) and heavy (2CD_3_) dimethyl groups. Then, the four different pseudo-isobaric labeled prepared samples were mixed in a 1:1:1:1 ratio and analyzed by MS^2^ scans with high resolution MS instrument to permitted relative quantification [202]. This approach may be employed for relatively high throughput and high accuracy comparative analysis of changes in plant proteome.

## Perspectives and conclusions

There is an increasing risk of yield reduction in agricultural crops due to biotic stress, and it is becoming increasingly important to elucidate how plants respond to biotic stress. Our basic understanding of plant responses to biotic stress needs to be further improved. An understanding of proteomics at the cellular and subcellular levels will provide precise regulation targets for plant immunity. As reviewed above, recent studies have revealed that intricate molecular mechanisms are involved in biotic stress. MS-based proteomic strategies allow measurement of proteins with critical functions and contributions to biotic stress mechanisms. However, many questions remain regarding plant proteomics and its application in biotic stress.

### At what depth can we cover the plant proteome?

To explain the molecular mechanism of protein level regulation in plants in response to biotic stress, it is important to know which proteins/proteomes are involved in this biological process, which raises the question of how many different proteins/proteomes can be identified in a tissue under certain conditions. Scientists have spent more than a decade studying how to increase proteome coverage in organisms, relying greatly on mass spectrometric and bioinformatic analyses. Although MS is a powerful method, there are limitations associated with the scanning rate of the mass spectrometer, incomplete databases and retrieval matching algorithms that make the identification and quantification of complete proteomes very challenging. For example, the scale of the human proteome remains a matter of debate. The number of potential protein-coding genes in the human genome is now estimated at ~ 20,000; however, the number of proteins in the human proteome ranges from 20,000 to several million [203–207]. To map the complete human proteome, thousands of LC–MS/MS analysis runs were combined, which have likewise mainly been attempted by very large-scale experiments [207–210]. Combined results showed that protein identification and quantification with high confidence at protein existence level 1 [211] has been reported in the range of 13,664–17,008, representing at least 70% of protein-coding genes in humans [204, 210, 212]. However, after decades spent increasing the metrics of proteome coverage, this aspect has not been analyzed systematically for plant proteome maps, with only 50% coverage achieved in Arabidopsis [213]. Therefore, it is necessary to quantitate and integrate the analysis of the broad-scale plant proteome. Effective analytical methods that identify proteins have advanced to provide high-quality information in human tissues, which are worthy of our study and use for reference in plant proteomics.

A typical whole-cell proteome contains more than 10,000 different proteins, with an abundance range of seven orders of magnitude, warranting further research on the whole-cell proteome [209, 214]. In particular, offline peptide fractionation at high pH reversed-phase chromatography, followed by peptide loading for low-pH online analysis in an LC/LC–MS system, has revealed numerous profiling data at the protein level in recent years [215]. Shotgun proteomic studies have identified ~ 12,200 proteins with prior multidimensional fractionation strategies [216]. The development of MS-based proteomics coupled with subcellular fractionation protocols has provided new opportunities to elucidate mechanisms under different conditions, such as disease and stress. To obtain a comprehensive view of the plant proteome, multiple techniques applied in human or animal models could be used in concert because of the advantages and shortcomings inherent to each method.

### How accurate are the proteomic data that we have?

MS-based proteomics is a highly complex analytical workflow and can be subject to large variability, leading to challenges in the acquisition of accurate and reproducible results. The results obtained from proteomics are limited by each phase: (1) sample preparation, including extraction and proteolytic digestion of the proteins; (2) peptide separation through LC; (3) MS analysis; and (4) informatic data interpretation. All these steps can introduce significant variability that needs to be accounted for at each step of the process from experiment to data analysis to obtain reproducible results. Consequently, a comprehensive and unbiased tool kit (standard, quality control or/and software) is a prerequisite for minimizing the existing variability and obtaining high-confidence results from different mass spectrometers and/or laboratories. Multiple workflows have been optimized that address these issues.

In the context of sample preparation, plant tissues often contain large amounts of carbohydrates, lipids, organic acids and many secondary metabolites, which have certain effects on protein extraction [217]. To overcome these problems, the method of protein precipitation by trichloroacetic acid (TCA)/acetone has been applied [218, 219]. TCA/acetone not only precipitates proteins but also dissolves a large number of contaminants, which improves the efficiency of protein extraction. In addition, some efficient and reproducible sample preparation strategies can also be used. Such as filter-aided sample preparation (FASP) [220], single-pot, solid-phase-enhanced sample preparation (SP3) [221] and the in-stagetip method (iST) [222]. However, the characteristics of all three approaches should be considered when using these three methods; the performance can differ in terms of proteome coverage as well as precision and reproducibility [223]. As a result, there is no single workflow that broadly fits all the sample processing in proteomics, and an optimal processing workflow for plant proteome analysis would ideally be built on the characterization of plant tissues or whole plants. There is no doubt that the rapid and deep proteomic analysis in plants will be essential in further research.

Another challenge associated with the reliability of proteomics results is data analysis. Given the large amount of available mass spectral data, the ability of data analysis directly determines whether valuable information can be obtained. Qualitatively, data analysis mainly refers to proteomic database searching. Currently, the main method of shotgun proteomics data analysis is to identify proteins by matching theoretical protein sequence databases with database search algorithms. The proteomic data resolution rate has increased from 50 to 85% [224]. The two main challenges that have been focused on are the proper matching characteristics of peptides and the spectra and credibility of the peptide matching results. Another challenge is data processing speed. In this respect, many representative studies have been conducted, such as studies using MaxQuant [225], Open-pFind [224], and MSFragger [226]. Moreover, software for top-down proteomics have also been used [227, 228]. Based on the statistical approach taken, or from a semiquantitative perspective, proteomics data analysis lacks a perfect decision-making process and is prone to misleading and incorrect data analysis and interpretation. To solve this problem, Marta et al. [229] analyzed and compared available methods of proteomics data analysis, providing a reference for other researchers.

### What are the other notable aspects of spatial and PTMs proteomics?

The identification of new targets and key regulators of stress remains an important but challenging goal for biotic stress research. MS-based proteomics has advanced and is now a versatile method that enables the generation of large datasets, including data regarding sequences, states of modification, quantities, protein structures and macromolecular contexts [230]. Several studies have shown that approximately 50% of the human proteome is located in multiple cellular compartments [207], which indicates that these proteins have multiple roles. These proteins may have two or more different cellular functions, depending on their subcellular environment [231]. The number of such proteins continues to increase [232]. In addition, proteins may change their position depending on cell cycle stages, circadian rhythms, or various stress factors [18]. These localization details need to be considered when designing spatial proteomic methods and validation experiments.

PTMs are key to many cellular signal transduction events, so it is important to know which proteins can be post-translationally modified and at which amino acid residue. In the case of phosphorylation, it remains unclear how many proteins are phosphorylated in the eukaryotic proteome and how many phosphorylation sites there are, despite the large number of high-throughput phosphoproteomic data that have emerged in the past decade [233, 234]. There is less phosphorylation data for Arabidopsis than for humans, mice and yeast [235]. Furthermore, in vivo, various kinds of PTMs do not exist in isolation, and the mutual coordination and influence of these PTMs enable complex life activities to proceed smoothly. Coordination of multiple PTMs ensures rapid and fine-tuned signal transduction and, in many cases, mediates activation and attenuation of the same pathway [236]. Collaborative studies on these kinds of multi-PTMs is emerging in the field of plant research. Therefore, there clearly remains much to be studied in plant PTMs research.

As emphasized above, MS-based proteomics has attracted many researchers with the ability to decipher complex proteomic networks and provides a holistic view at the molecular level that may better reflect the phenotype of plant resistance. If carefully designed and validated, spatial proteomics and PTMs proteomics, with appropriate bioinformatic assistance, are invaluable tools that make it possible to uncover plant resistance mechanisms. We are optimistic that an increasing number of laboratories will adopt, practice and advance MS-based proteomics to answer important biological questions.

## Data Availability

Not applicable.

## Abbreviations

PTMs: post-translational modifications
LC–MS: liquid chromatography-mass spectrometry
FFE: free flow electrophoresis
FIFFF: flow field-flow fractionation
LCM: laser capture microdissection
ABPP: activity-based protein profiling
APEX: ascorbate peroxidase
ORMs: organelle-locatable reactive molecules
BioID: proximity-dependent biotin identification
IMAC: immobilized metal affinity chromatography
MOAC: metal oxide affinity chromatography
HILIC: hydrophilic interaction liquid chromatography
ICAT: labeling-based strategies include isotope-coded affinity tag
iTRAQ: isobaric tags for relative and absolute quantification
TMT: tandem mass tag
AACT/SILAC: amino acid-coded mass tagging/stable isotope labeling with amino acids in cell culture
QconCAT: uniform 15N/18O labeling and peptides or quantitative concatemer
SWATH: sequential windowed acquisition of all theoretical fragment ion mass spectra
MdFDIA: mass defect-based four-plex data-independent acquisition strategy
CTAP: cell-selective labeling with amino acid precursors
ICAT: isotope-coded affinity tag
ICPL: isotope-coded protein labeling
DiLeu: *N*,*N*-dimethyl leucine
IPTL: isobaric peptide termini labeling
OGs: oligogalacturonides
WAK1: wall-associated kinase 1
CWPs: cell wall proteins
DIR: dirigent proteins
PAMPs: pathogen- associated molecular patterns
MAMPs: microbe- associated molecular patterns
DAMPs: danger-associated molecular patterns
PTI: pattern-triggered immunity
ETI: effector-triggered immunity
RLKs: receptor-like kinases
RLPs: receptor-like proteins
PRRs: pattern recognition receptors
FLS2: flagellin sensing2
BAK1: brassinosteroid insensitive1-associated kinase1
flg22: a 22-amino-acid epitope
CRKs: Cys-rich receptor-like kinases
SERK: somatic embryogenesis receptor kinase
PCD: programmed cell death
mETC: mitochondrial electron transport chain
ROS: reactive oxygen species
OEE: oxygen evolving enhancer
MPP: mitochondrial processing peptidase
HSP: heat shock protein
SA: salicylic acid
JA: jasmonic acid
JAZ7: jasmonate ZIM-domain 7
Pst. DC3000: *Pseudomonas syringae pv. tomato* DC3000
ABA: abscisic acid
AHL: AT-hook-like
EVs: extracellular vesicles
RIN4: RPM1-interacting protein4
EFR: EF-Tu receptor
elf18: EF-Tu epitopes
SERK3: somatic embryogenesis receptor kinase 3
BIK1: botrytis-induced kinase 1
PP2A: protein phosphatase 2A
Co-IP: coimmunoprecipitation
TTSS: type III secretion system
Lys: lysine residue
E1: ubiquitin-activating enzyme
E2: ubiquitin-conjugation enzyme
E3: ubiquitin ligase
SUMO: small ubiquitin-like modifier
SAE: SUMO-activating enzyme
SCE: SUMO-conjugating enzyme
NATs: Nt-α-acetyltransferases
NLRs: Nod-like receptors
NPR1: natriuretic peptide receptor A
NATB: NAT complex B
HATs: histone acetyltransferases
HDACs: histone deacetylases
MALDI-TOF MS: matrix-assisted laser desorption ionization-time-of-flight MS
FASP: filter-aided sample preparation
SP3: single-pot, solid-phase-enhanced sample preparation
iST: in-stagetip method
VIC: vacuum infiltration-centrifugation
